# 98% IGHV gene identity is the optimal cutoff to dichotomize the prognosis of Chinese patients with chronic lymphocytic leukemia

**DOI:** 10.1002/cam4.2788

**Published:** 2019-12-17

**Authors:** Ke Shi, Qian Sun, Chun Qiao, Huayuan Zhu, Li Wang, Jiazhu Wu, Lili Wang, Jianxin Fu, Ken H. Young, Lei Fan, Yi Xia, Wei Xu, Jianyong Li

**Affiliations:** ^1^ Department of Hematology the First Affiliated Hospital of Nanjing Medical University Jiangsu Province Hospital Nanjing China; ^2^ Key Laboratory of Hematology of Nanjing Medical University Nanjing China; ^3^ Department of Hematology The First People's Hospital of Yancheng The Forth Affiliated Hospital of Nantong University Yancheng China; ^4^ Collaborative Innovation Center for Cancer Personalized Medicine Nanjing China; ^5^ Department of Systems Biology Beckman Research Institute and NCI City of Hope Comprehensive Cancer Center Duarte CA USA; ^6^ Hematopathology Division and Pathology Department Duke University School of Medicine Duke Medical Center and Cancer Institute Durham NC USA

**Keywords:** Chinese, chronic lymphocytic leukemia, cutoff value, immunoglobulin heavy chain variable region, prognosis

## Abstract

Immunoglobulin heavy chain variable region (IGHV) mutational status has been an important prognostic factor for chronic lymphocytic leukemia (CLL) for decades. Patients with unmutated IGHV (≥98% identity to the germline sequence) have inferior prognosis and tend to carry unfavorable genetic markers compared to those with mutated IGHV (<98% identity to the germline sequence). However, 98% as the cutoff for IGHV mutational status is a mathematical choice and remains controversial. We have previously reported distinct IGHV repertoire features between Chinese and western CLL populations. Here, we retrospectively studied 595 Chinese CLL patients to determine the best cutoff value for IGHV in Chinese CLL population. Using 1% as the interval for IGHV identity, we divided the studied cohort into seven subgroups from 95% to 100%. Briefer time to first treatment (TTFT) and overall survival (OS) were observed in cases with ≥98% compared to those with <98%, while the differences were obscure within subgroups ≥98% (98%‐98.99%, 99%‐99.99%, and 100%) and <98% (<94.99%, 95%‐95.99%, 96%‐96.99%, and 97%‐97.99%). Multivariate analysis confirmed the independent prognostic value of 98% being the cutoff for IGHV identity in terms of both TTFT and OS. All the prognostic factors, including del(17p13), del(11q22.3), *TP53* mutation, *MYD88* mutation, *NOTCH1* mutation, *SF3B1* mutation, CD38, ZAP‐70, Binet staging, gender, and β2‐microglobulin, were significantly different in distribution between group <98% and group ≥98%, but not among subgroups 98%‐98.99%, 99%‐99.99%, and 100%. In conclusion, 98% is the optimal cutoff of IGHV identity for the prognosis evaluation of Chinese CLL patients.

## Graphical abstract

In this article, we focused on whether 98% is the best cutoff value for IGHV in Chinese patients on a 595 CLL cohort with 600 sequences in total. We found that for the whole cohort, 98% showed to be the best cutoff value for IGHV, holding prognostic value for both time to first treatment (TTFT) and overall survival (OS) in univariate analysis and being an independent prognostic factor for TTFT and OS in multivariate analysis. At the same time, all the prognostic factors were also significantly different in distribution between group <98% and group ≥98%, while the distributions among subgroups ≥98% were not significantly different. We proved that 98% IGHV is an optimal choice to predict the prognosis of CLL patients in China at present.

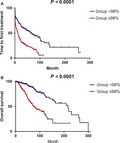

## 1 INTRODUCTION

Chronic lymphocytic leukemia (CLL) is the most common type of adult leukemia in western countries.^1^ The mutational status of the immunoglobulin heavy chain variable region (IGHV) gene is one of the most important prognostic factors for CLL patients with high identity to the germline IGHV sequence corresponding to poor prognosis.^2^, ^3^, ^4^, ^5^, ^6^, ^7^, ^8^, ^9^ The criteria for differentiating IGHV mutational status varied from 95% to 98%. The 98% cutoff value to dichotomize IGHV mutational status recommended by European Research Initiative on CLL (ERIC) has been widely used nowadays^10^: IGHV sequences with <98% identity to the germline sequence are termed as mutated (M), while those with ≥98% identity to the germline sequence are termed as unmutated (UM). However, the “borderline” cases defined as 97%‐97.99% identity to the germline represent a mixture group of both indolent and aggressive cases,^10^, ^11^ which brings about discussions regarding the optimal cutoff value for IGHV. Davis et al proved that trichotomy by 97% and 99% could better predict time to first treatment (TTFT) (in stage A cohort) and overall survival (OS) for the whole cohort (after ruling out cases with subset#2). Progression‐free survival (PFS) was also different between <97% cohort and ≥97% cohort in the clinical trial cohort.^12^ Another team proved that continuous IGHV mutational rate rather than a point value was prognostically significant in patients treated with fludarabine, cyclophosphamide, and rituximab (FCR).^13^

Since all of the previous researches on IGHV cutoff values were from western countries predominantly composed of Caucasian, who are different from Asian CLL patients in terms of both clinical features and IGHV gene usage,^14^, ^15^ we conducted this retrospective study including 595 cases in order to fill the gap of IGHV cutoff value study in Chinese CLL patients. Through clinical correlation and survival analysis, we found that 98% IGHV gene identity is still the optimal choice for prognosis prediction in our study cohort.

## 2 MATERIALS AND METHODS

### 2.1 Patients

The study includes 595 patients with newly diagnosed CLL/small lymphocytic leukemia (SLL) based on the criteria by the International Workshop on CLL‐National Cancer Institute (IWCLL‐NCI).^16^ All patients were from our center (diagnosed from 2002 to 2017). The study was approved by the Ethics Committee of the First Affiliated Hospital of Nanjing Medical University with a reference number 2014‐SR‐204. Informed consents were provided according to the Declaration of Helsinki before the samples were collected.

### 2.2 Analysis of immunoglobulin rearrangements

Mononuclear cells were isolated from peripheral blood by lymphocytes separation medium. Then gDNA or cDNA was subjected to polymerase chain reaction (PCR) amplification following the IGH Somatic Hypermutation Assay v2.0 protocol (InVivoScribe) (PCR was performed by Veriti 96‐well thermal cycler, applied biosystems). The kit provides both leader primers and FR1 primers. The later was used in case of failed detection after using leader primers. In most cases, we used the leader primers to determine the IGHV somatic hypermutation status of clonotypic IGHV‐IGHD‐IGHJ gene rearrangements. IGHV*‐*D‐J rearrangements were sequenced by 3130 Genetic Analyzer (Life Technologies, Carlsbad, CA).

Sequences were aligned to ImMunoGeneTics/V‐QUEry and Standardization (IMGT/‐VQUEST) database and the IMGT/V‐QUEST tool (version 3.3.0). IGHV usages and rates of somatic hypermutation of productive rearrangements were recorded. Adjusted IGHV identity only happened when the option “search for insertions/deletions” was shown.

### 2.3 Cytogenetics and analyses of *TP53*, *NOTCH1*, *SF3B1*, and *MYD88* mutations

We performed interphase fluorescence in situ hybridization (FISH) of del(11q22.3), del(17p13), del (13q14), and trisomy 12 and Sanger sequencings of *TP53* (exon 4‐9), *NOTCH1* (PEST domain), *SF3B1* (exon 14‐16), and *MYD88* (exon 3‐5), as described previously.^17^, ^18^

### 2.4 Immunophenotyping

The procedures of immunophenotyping of CD38 and ZAP70 by flow cytometry were described previously.^17^ The positive cutoff values for CD38 and ZAP‐70 were 30% and 20%, respectively.

### 2.5 Statistical analysis

OS was calculated from diagnosis to death or last follow‐up. TTFT was calculated as time between diagnosis and first treatment. Survival curve was generated via the method of Kaplan‐Meier. Log‐rank test was used for significant associations. Categorical variables were compared by Chi‐square test. Cox regression analysis was constructed to determine the hazard ratio (HR). Variables of significance in univariate analysis were included in multiple Cox proportional hazards model. Statistical analyses were performed by IBM SPSS Statistics 23 (IBM Corporation, Armonk, NY, USA). Tables and figures were drawn by Microsoft office 2016 software and Graphpad Prism 7.0 (GraphPad Software, San Diego, CA) software. *P*‐values were two‐sided and *P* values < 0.05 were considered significant.

## 3 RESULTS

### 3.1 Subjects

The characteristics of 595 patients were summarized in Table 1. The median age was 61.4 years (range 16‐92) and 60.9% were male. Twenty‐eight patients were diagnosed with SLL and the rest of 567 were CLL patients. 214 (39.9%) patients were in Binet A, 148 (25.8%) in Binet B, and 184 (34.3%) in Binet C. Sixteen (2.7%) patients suffered from Richter's syndrome.

**Table 1:** Clinical and immunogenetic characteristics of Chinese CLL patients at diagnosis

Variables	N = 595
Age	61.4 (16‐92)
Male	380 (63.9%)
Binet stage (n = 536)	
A	214 (39.9%)
B	138 (25.8%)
C	184 (34.3%)
White blood cell count (×10^9^/L) (n = 522)	42.6 (5.7‐426.2)
Absolute lymphocyte count (×10^9^/L) (n = 522)	36.1 (3.5‐416.2)
Hemoglobin (g/L) (n = 522)	120 (26‐196)
Platelet (×10^9^/L) (n = 522)	139 (4‐477)
IGHV unmutated	248 (41.3%)
β2‐microglobulin (mg/L) (n = 490)	3.99 (1‐22)
CD38 positive (n = 572)	175 (31%)
ZAP70 positive (n = 566)	222 (40%)
Hepatitis B virus positive (n = 462)^a^	41 (8.9%)
Molecular abnormalities	
*TP53* mutation (n = 490)	70 (14.3%)
*SF3B1* mutation (n = 413)	24 (5.8%)
*NOTCH1* mutation (n = 465)	34 (7.3%)
*MYD88* mutation (n = 409)	32 (7.8%)
FISH	
Del (17p13) (n = 507)	75 (14.8%)
Del (11q22.3) (n = 486）	77 (15.8%)
Trisomy 12 (n = 429)	103 (24%)

a We defined Hepatitis B virus positive as positive for surface antigen and/or HBV‐DNA positive.

After a median follow‐up of 45 months (range 1‐279 months), 359 (61.8%) patients were treated (median numbers of therapies: 2). Three hundred and four patients had unequivocal initial treatment information as follows: 115 (37.8%) patients received rituximab‐based therapy (rituximab alone or immunochemotherapy), 181 (59.5%) patients received chemotherapy alone, five (1.6%) patients received ibrutinib as single agent, and three (0.99%) patients received other therapies (one patient treated with decitabine, two patients treated with high‐dose methylprednisolone).

### 3.2 IGHV usage

**Figure 1:**
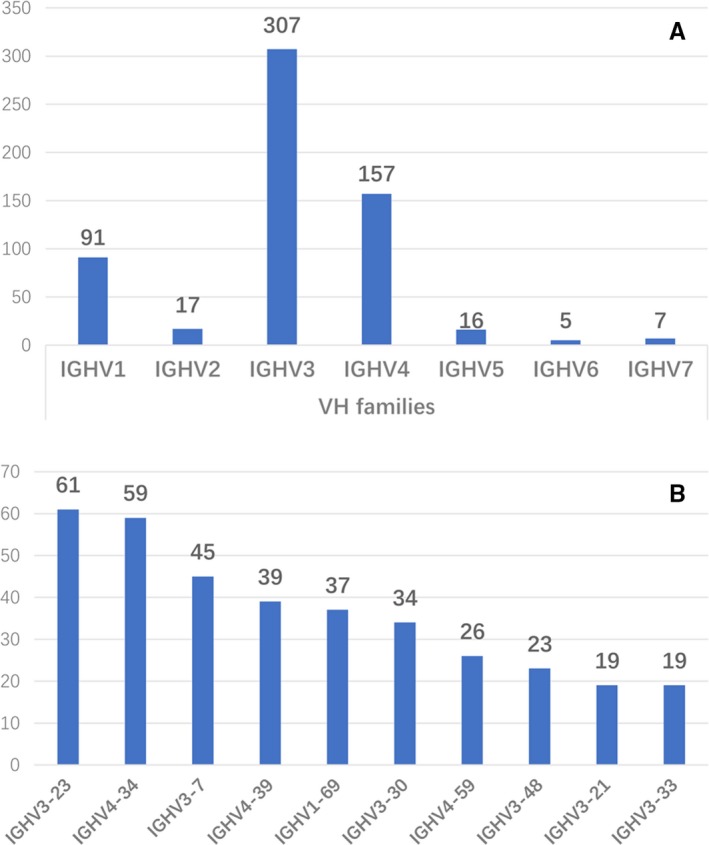
The biased used IGHV families and IGHV subgroups in Chinese CLL patients. A, IGHV3 (51.2%) was the most frequently used family, followed by IGHV4 (26.2%), IGHV1 (15.2%), IGHV2 (2.8%), IGHV7 (1.2%), and IGHV6 (0.8%). B, The most frequent IGHV subgroups were IGHV3‐23 (61, 10.7%), IGHV4‐34 (59, 9.83%), IGHV3‐7 (45, 7.5%), IGHV4‐39 (39, 6.5%), IGHV1‐69 (37, 6.17%), IGHV3‐30 (34 5.67%), IGHV4‐59 (26, 4.33%), IGHV3‐48 (23, 3.83%), IGHV3‐21 (19, 3.17%), and IGHV3‐33 (19, 3.17%)

IGHV‐D‐J sequencing was conducted on all the 595 patients. A total of 600 sequences were obtained as five (0.8%) patients held double rearrangements (Table S1). With the exception of Patient 4, the mutational rates of the two sequences in the rest of the patients were similar. We included both sequences of each patient in subsequent analyses. Six hundred sequences were unevenly distributed among seven IGHV families, showing the overuse of IGHV3 (51.2%), followed by IGHV4 (26.2%), IGHV1 (15.2%), IGHV2 (2.8%), IGHV7 (1.2%), and IGHV6 (0.8%) (Figure 1A). The most frequent IGHV subgroups were IGHV3‐23 (61, 10.7%), IGHV4‐34 (59, 9.8%), IGHV3‐7 (45, 7.5%), IGHV4‐39 (39, 6.5%), IGHV1‐69 (37, 6.2%), IGHV3‐30 (34, 5.7%), IGHV4‐59 (26, 4.3%), IGHV3‐48 (23, 3.8%), IGHV3‐21 (19, 3.2%), and IGHV3‐33 (19, 3.2%) (Figure 1B).

### 3.3 Influence of mutational load on clinical outcomes

**Figure 2:**
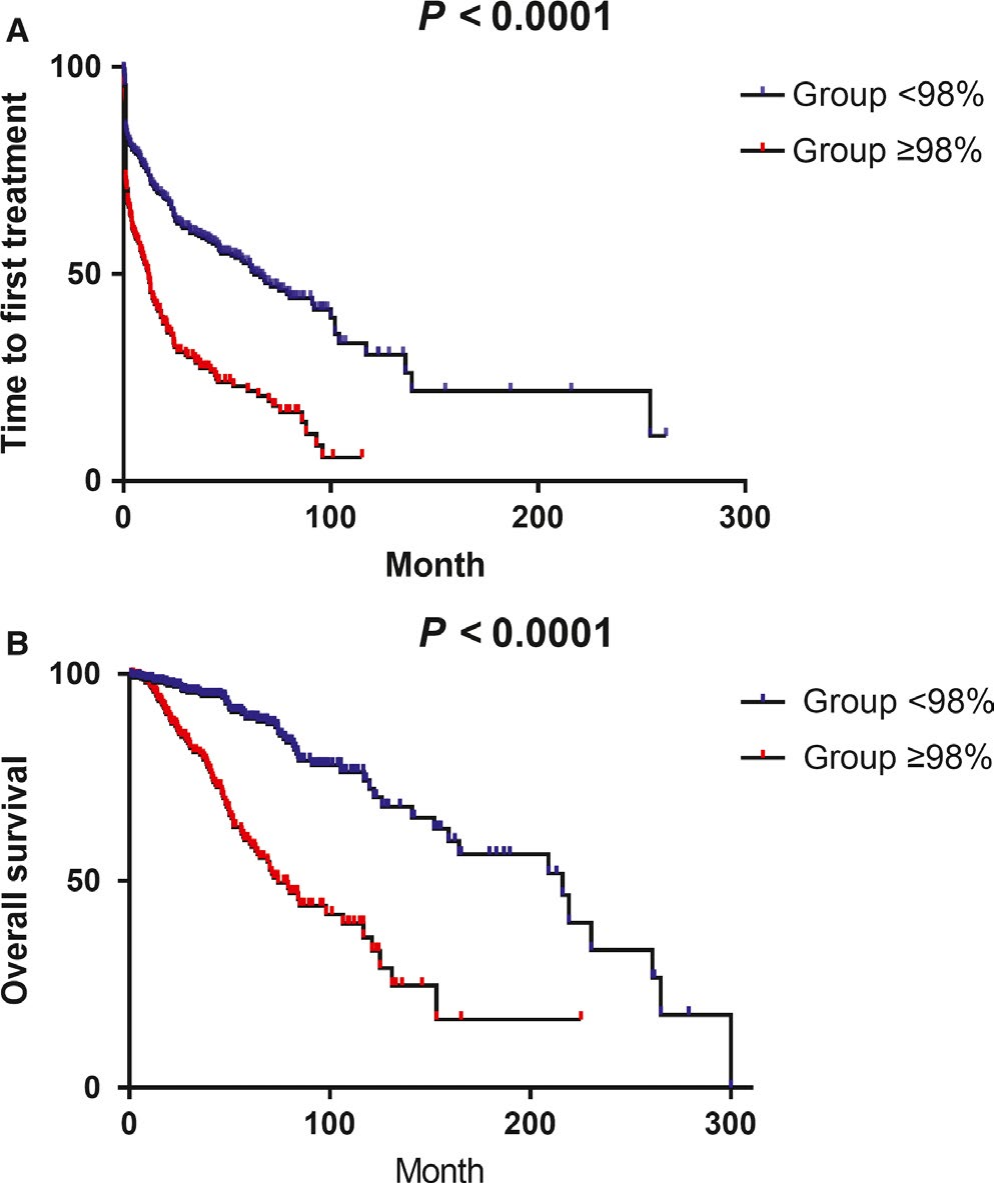
Survival differences between IGHV (98% as the cutoff value) subgroups (both *P* value < 0.0001). Five hundred and eighty‐six patients were included in these analyses. A, TTFT between IGHV subgroups. Median TTFT: 61.9 months for < 98% group while 9 months for ≥ 98% group. B, OS differences between IGHV subgroups. Median OS: 216 months for < 98% group while 74 months for ≥ 98% group

About 352 (58.7%) cases were M, while 248 (41.3%) cases were UM if we used the classical 98% classification by ERIC. In order to determine the optimal cutoff value, we used 1% as the interval to divide the entire cohort into seven groups according to the mutational rate, which were <95%, 95%‐95.99%, 96%‐96.99%, 97%‐97.99%, 98%‐98.99%, 99%‐99.99%, and 100%, respectively. First, we investigated the best cutoff value in Binet A patients (n = 213，with one patient lost to follow‐up). Cox regression analysis showed that only the 100% group (hazard ratio (HR): 2.46, *P* = .001) was significantly different in TTFT when compared with the <95% group (Table 2A). Then, we compared TTFT in the whole cohort (n = 586，with nine patients lost to follow‐up). Significant difference appeared at the 98% interval with a HR of 2.3 (*P* < .001), while intervals less than 98% had no significant difference compared with the <95% group (HR: 0.93 for subgroup 95%‐95.99%, 0.78 for subgroup 96%‐96.99%, 1.37 for subgroup 97%‐97.99%, Figure 2A). Similarly, there were no clear dissimilarities in HR among the 98%‐98.99%, 99%‐99.99%, and 100% subgroups which were 2.3, 2.9, and 2.34, respectively (Table 2B). The same 98% cutoff was also desirable in the OS prediction for the whole study cohort (Figure 2B, only 23 cases in the Binet A cohort died, failing to evaluate OS): comparing to the < 95% group, patients in 95%‐95.99%, 96%‐96.99%, and 97%‐97.99% subgroups tended to have lower HR (0.5‐1.02) while patients in 98%‐98.99%, 99%‐99.99% and 100% subgroups had significantly higher HR (2.94, 3.44 4.25, respectively, Table 2C).

**Table 2:** Cox remission analyses of (A) TTFT in the Binet A cohort, TTFT in the whole cohort, and (B) OS in the whole cohort (HR: Hazard ratio; 95% CI: 95% confidence interval

IGHV% identities	Numbers	Mean (month)	Median (month)	HR	95% CI	*P*
(A) Cox remission of TTFT in the Binet A cohort						
TTFT						
<95%	113	134.7	139	1.00		
95%‐95.99%	19	85.2	100	0.57	(0.21,1.61)	.29
96%‐96.99%	17	96.0	Not reached	0.34	(0.08,1.41)	.14
97%‐97.99%	9	91.8	Not reached	1.17	(0.42,3.31)	.76
98%‐98.99%	10	60.8	Not reached	1.55	(0.55,4.35)	.41
99%‐99.99%	9	53.6	88	2.03	(0.80,5.16)	.14
100.00%	36	49.6	34.5	2.46	(1.44,4.21)	.001*
(B) Cox remission of TTFT in the whole cohort						
TTFT						
<94.99%	230	99.4	58	1.00		
95%‐95.99%	46	66.3	92	0.93	(0.59,1.47)	.75
96%‐96.99%	36	66.2	71	0.78	(0.45,1.34)	.37
97%‐97.99%	31	57.2	19	1.37	(0.85,2,2)	.19
98%‐98.99%	37	27.7	13	2.30	(1.53,3.48)	<.001*
99%‐99.99%	64	20.6	4	2.90	(2.08,4.06)	<.001*
100.00%	142	28.5	11	2.34	(1.79,3.06)	<.001*
(C) Cox remission of OS in the whole cohort						
OS						
<94.99%	230	195.5	230.3	1.00		
95%‐95.99%	46	120.6	119.7	1.02	(0.43,2.42)	.97
96%‐96.99%	36	191.3	219.1	0.50	(0.15,1.62)	.25
97%‐97.99%	31	131.8	Not reached	0.83	(0.30,2.34)	.73
98%‐98.99%	37	113.2	98	2.94	(1.30,4.42)	.005*
99%‐99.99%	64	93.5	106.5	3.44	(2.05,5.78)	<.001*
100.00%	142	82.1	69	4.25	(2.78,6.5)	<.001*

* *P* values < 0.05 and with significant difference.

It has been reported that up to 30% of CLL patients belong to B cell receptor (BCR) stereotypy and with “some” subsets conferring specific clinical outcomes, especially those who belong to subset#2 characterized with IGHV3‐21 usage and predominantly mutated IGHV status.^11^, ^19^, ^20^ Lacking the CDR3 information in 131 patients limited further identification of subsets. However, we still identified subsets of the remaining 469 sequences. There was only one patient belonging to subset#2 in 469 evaluable sequences and the result was consistent with our previous study.^15^ Since the rarity of subset#2 in our research, we thought the isolated case could not affect the result of cutoff analysis, so we reached a compromise by excluding all the IGHV3‐21 cases that tend to have poor prognosis, though there have been controversies over the prognosis of them.^21^, ^22^ 98% cutoff value could still better predict TTFT and OS in cohort without IGHV3‐21 cases than any other cutoff values (Table S2).

### 3.4 Clinical correlations

Although 98% was the appropriate cutoff for TTFT and OS in our study, we still wanted to know if there were a maldistribution of other prognostic factors in the M/UM groups and whether it was due to the increase of these poor prognostic factors that led to a gradual increase in the HR of OS among three intervals that are ≥98% (2.94, 3.44, 4.25, respectively).

If we dichotomized the whole cohort by 98%, patients in ≥98% group were more likely to be male (*P* = .009), with higher levels of β_2_‐MG at diagnosis (*P* < .001) and in advanced Binet stages (*P* < .001). The distribution of *TP53*, *NOTCH1*, *SF3B1*, and *MYD88* mutations was significantly different (*P* < .001, *P* < .001, *P* = .034, and *P* < .001, respectively), so was the distribution of del(17p13), del(11q22.3), CD38^+^, and ZAP‐70^+^ (*P* = .01, *P* < .001, *P* < .001, and *P* = .005, respectively, Table 3).

**Table 3:** The relationships between various factors and TTFT/OS; the distributions of factors among intervals

	Numbers	TTFT	OS	Distributions between <98% and ≥98%	Distributions among 98%‐98.99%, 99%‐99.99%, and 100%
Del(17p13)	504	<0.001	<0.001	0.01	×
*TP53*	485	<0.001	<0.001	<0.001	×
Del(11q22.3)	481	<0.002	0.169#	<0.001	×
Trisomy 12	429	0.443*	0.254*	0.47*	×
*MYD88*	409	0.715*	0.087*	<0.001	×
*NOTCH1*	465	<0.001	<0.001	<0.001	×
*SF3B1*	413	0.054#	0.056#	0.034	✓
CD38	569	<0.001	0.024	<0.001	×
ZAP‐70	564	0.036	0.2#	0.005	×
β2‐MG	493	<0.001	<0.001	<0.001	×
Binet stage	534	<0.001	<0.001	<0.001	×
Gender	586	0.01	0.01	0.009	×
HBV	462	0.978*	0.439*	0.216*	×
Age	586	0.872*	0.351*	0.283	×

* Without significant difference.

^#^ Without significant difference but *P* ≤ .2.

^×^ Distributions among the three intervals were not significantly different.

^✓^ Distributions among the three intervals were significantly different.

There were no statistically significant differences in the distribution of these prognostic factors in the three subgroups that are ≥98% with the exception of *SF3B1* mutation. The frequency of *SF3B1* mutation in the 98%‐98.99% group was significantly higher than that in the 99%‐99.99% and 100% groups (*P* = .032, *P* = .004, respectively, Table 3). But given that *SF3B1* mutation was not a prognostic factor for TTFT and OS in our study, it did not change our conclusion. On the other hand, due to the low mutation rate of *SF3B1*, theoretical frequencies were less than 5 in some groups.

Therefore, we conclude that within the ≥98% group, the gradual increase in HR was more likely due to the decrease in IGHV mutational rate rather than other concurrent effects of poor prognostic factors we have known. The lower the mutational rate of IGHV is, the higher the HR of OS is.

### 3.5 Multivariate analyses

In univariate analyses, we found out that clinical features (Binet staging, gender, and β_2_‐microglobulin (β_2_‐MG)>3.5 mg/L), cytogenetic aberrations (*TP53* mutations, *NOTCH1* mutations, and del(17p13)), and immunophenotyping (CD38 positive and ZAP‐70 positive) were prognostic factors for both TTFT and OS. Del(11q22.3) was the prognostic factor for TTFT.

Then we conducted Multivariate Cox regression analyses containing prognostic factors above. UM‐IGHV (HR: 1.68; 95% CI: (1.26,2.25); *P* < .001) and advanced Binet stages (stage B: HR: 4.04; 95% CI: (2.85,5.74); *P* < .001; stage C: HR: 3.10; 95% CI: (2.14,4.49); *P* < .001) were independently correlated with TTFT. UM‐IGHV (HR: 2.30; 95% CI: (1.36,3.89); *P* = .002), advanced Binet stages (stage B: HR: 2.68; 95% CI: (1.41,5.12); *P* = .003), the presence of *TP53* mutations (HR: 1.87; 95% CI: (1.07,3.28); *P* = .029), del(17p13) (HR: 2.26; 95% CI: (1.21,4.19); *P* = .01), *NOTCH1* mutations (HR: 2.21; 95% CI: (1.15,4.25); *P* = .017), and male (HR: 1.77; 95% CI: (1.06,2.95); *P* = .03) were independently correlated with OS (details in Table 4). β_2_‐MG showed marginal significance in multivariate analysis of TTFT (*P* = .053). It should be noted that neither *TP53* mutation nor del(17p13) was independent prognostic factors for TTFT in this cohort, probably due to their weak power as indications for treatment of CLL.

**Table 4:** Multivariate analyses of TTFT and OS in the whole cohort (showing the independent prognostic factors)

Variables	*P*	HR	95% CI
(A) TTFT*			
98% as cutoff value	<0.001	1.68	(1.26,2.25)
Binet A	<0.001		
Binet B	<0.001	4.04	(2.85,5.74)
Binet C	<0.001	3.10	(2.14,4.49)
(B) OS*			
98% as cutoff value	0.002	2.30	(1.36,3.89)
Binet A	0.01		
Binet B	0.03	2.68	(1.40,5.12)
Binet C	0.09	1.86	(0.91,3.80)
TP53 mutations	0.029	1.87	(1.07,3.29)
NOTCH1 mutations	0.017	2.22	(1.15,4.25)
Del(17p13)	0.01	2.26	(1.21,4.19)
Gender	0.01	1.77	(1.06,2.95)

* Totally 305 patients were available in analysis*.*

## 4 DISCUSSION

Initially, Damle et al and Hamblin et al chose 98% as the cutoff value mainly based on statistical results.^5^, ^6^ No wonder other cutoff values, such as 95% and 97%, had appeared in succession. Studies on origin and differentiation of CLL theoretically supported the feasibility of statistically selecting 98% as the cutoff value: cells going through T‐cell‐independent pathway in marginal zones usually have no or limited IGHV gene mutations (clinically UM cases), while cells going through T‐cell‐dependent pathway in germinal centers undergo IGHV gene mutations (clinically M cases) with the influence of microenvironment.^23^ M cases and UM cases show variant prognosis with the former having the characteristics of memory B lymphocytes and the latter resemble naïve B lymphocytes.^23^, ^24^ Also, M CLL cells seem to hold longer telomeres than UM ones.^25^ Some studies argued that from the perspective of molecular biology, the grouping of CLL patients based on IGHV mutational status could be further refined. Degan M et al believed that based on the antigen‐driven selection, CLL patients could be divided into three subgroups: significantly mutated, not significantly mutated, and UM.^26^ Another epigenetic research on DNA methylation analysis also grouped CLL into three subgroups: naïve B cell‐like CLL (resembling UM CLL), intermediate CLL, and memory B cell‐like CLL. Although authors demonstrated that the latter two belonged to M CLL, intermediate CLL (mean load of IGHV mutation: 96.7%) was a relatively complex group with biased use of IGHV1‐18 and higher rate of *SF3B1* mutation.^27^ Perhaps the current debate on the best IGHV cutoff values was due to the effect of this group of cells with complex performance.

There are differences in IGHV and BCR stereotypy usage between CLL patients from East Asia and those who come from western countries. IGHV1‐69, IGHV3‐07, IGHV3‐23, and IGHV4‐34 are the most frequently used genes in CLL patients from the West, while IGHV4‐34, IGHV3‐23, IGHV3‐07, and IGHV4‐39 are the most frequently used genes in patients from East Asia.^4^, ^5^, ^6^, ^14^, ^15^ In western CLL patients, almost one third of the IGHV sequences belong to stereotyped BCRs. However, in East Asian patients, the proportion of stereotyped BCRs is significantly lower—in our previous research, the ratio is 22.4%.^14^, ^15^ Of note, patients in East Asia seem to have a significantly higher proportion of subset #8, while subset #2 common in western patients is rare.^15^ These differences within IGHV sequences between ethnic groups possibly originate from ethnic genetic diversity and environmental effects, which urged us to seek the best cutoff value for IGHV in Chinese patients.

In this study, we testified 98% is the optimal cutoff for IGHV in Chinese CLL patients for prognosis evaluation. At the same time, ERIC do also note that caution is warranted in borderline cases and that the clinical implications remain to be elucidated. Multivariate analysis showed that 98% cutoff value was an independent prognostic factor for TTFT and OS. All the prognostic factors involved in our study were significantly different in the two groups that were dichotomized at 98%, indicating high efficiency of 98% as a cutoff value for IGHV in assessing patients. We also found in the subgroups of ≥98%, the increased HR was consistent with an increased IGHV identity to germline sequence and not to other prognostic factors. However, the IGHV mutational status seemed to have limited effects on TTFT and OS in our Binet A cohort (accounting for 40% of the whole cohort), which may have resulted from the uneven distribution of numbers in each interval (the five groups of 95%‐99% had no more than 20 cases in each group). A larger CLL cohort is needed to verify this result.

Also, there were some limitations in our study. First of all, follow‐up time in our study was shorter compared with that in similar studies.^12^, ^13^ In addition, due to the heterogeneity of therapy, we did not take the effects of treatment into consideration in neither univariate nor multivariate analysis. Therefore, it cannot be completely ruled out that the increase in HR within the ≥98% group was partially affected by treatment strategies, while PFS was adopted as an important index for assessing the efficacy of treatment in the other two studies recently published regarding IGHV cutoff value.^12^, ^13^ Among them, Jain et al believed that IGHV as a continuous variable among patients treated with FCR can accurately predict the patient's PFS. We hoped for an appropriate Chinese patient cohort to explore whether PFS should be measured using different IGHV cutoff criteria.

In conclusion, we show that 98% cutoff value for IGHV is still the optimal choice for clinical applications. But nothing stays unchangeable. In the current “new agent” era, targeted drugs, such as ibrutinib, idelalisib, and venetoclax (ABT‐199), have affirmative effects on UM patients.^28^, ^29^, ^30^ When using ibrutinib in relapse/refractory UM patients, the median PFS was 43 months after a median follow‐up of 5 years.^31^ However, targeted drugs are generally expensive and the follow‐up time of clinical trials is not long enough. IGHV mutational status together with FISH could still better predict TTFT in newly diagnosed patients, which could guide on follow‐up time and treatment strategies.^30^ On the other hand, with the development of new technologies, such as next‐generation sequencing (NGS), up to 25% of CLL patients had ≥2 IGHV rearrangements (the ratio was only 5% by Sanger sequencing).^32^, ^33^ The phenomenon that one patient has multiple sequences grouped into different mutational status (according to current standards) also exists. Once NGS has been widely used, is 98% the optimal cutoff value for IGHV still appropriate, or is there a revolutionary subversion of the definition of IGHV mutational status? These hypotheses require further researches to be certified.

## CONFLICT OF INTEREST

The authors declare that they have no conflict of interest to disclose.

## AUTHOR CONTRIBUTIONS

JYL, W.X, Y.X, JXF, LLW, and KHY designed the study. K.S, Q.S, C.Q, HYZ, L.W, JZW, and L.F collected and analyzed the data. Q.S and K.S wrote the draft of the paper and all authors contributed to the writing and approved the final version of the manuscript.

## Data Availability

I confirm that my article contains a Data Availability Statement even if no data is available unless my article type does not require one. I confirm that I have included a citation for available data in my references section, unless my article type is exempt.
